# Evaluation of dose distributions and respiratory motion tolerance for layer-stacking conformal carbon-ion radiotherapy

**DOI:** 10.1007/s12194-024-00847-1

**Published:** 2024-11-14

**Authors:** Yuki Hasebe, Mutsumi Tashiro, Hiroshi Sakurai

**Affiliations:** 1 Graduate School of Science and Technology, Gunma University, 1-5-1 Tenjin-Cho, Kiryu, Gunma 376-8515 Japan; 2 Gunma University Heavy Ion Medical Center, 3-39-22 Showa-Machi, Maebashi, Gunma 371-8511 Japan

**Keywords:** Carbon-ion radiotherapy, Passive beam, Layer-stacking irradiation, Respiratory motion, Motion tolerance

## Abstract

While layer-stacking irradiation provides a conformal dose distribution, it is vulnerable to respiratory motion. Considering that the motion tolerance has not yet been demonstrated, this study aimed to determine the tolerance level for the amount of target motion. Dose distributions considering motion were simulated for a numerical water phantom using in-house software. Comparisons with measured and simulated physical dose distributions confirmed the validity of the simulation, with gamma analysis showing almost 90% or greater agreement under all conditions with a criterion of 3%/3 mm. The variation in physical dose from static conditions followed a similar trend. Based on the evaluation of the simulated clinical dose uniformity, motion tolerance was derived. The acceptable motion amounts in the lateral direction were 11 mm in respiratory-ungated condition and at least 20 mm with 30% lateral gating at 4 Gy (RBE). In the longitudinal (beam upstream) direction, the acceptable target motion amounts were 3 mm without gating and 6 mm with gating. These results employed worst-case scenarios considering multiple respiratory cycles. In both lateral and longitudinal directions, the motion amounts of 3 mm for non-gating and 5 mm for gating were acceptable. The acceptable target motion amounts improved by 1–9 mm with gating and increased prescribed doses. The dose uniformity and motion tolerance under multiple conditions, although based on a simple system, may be useful for treatment involving target motion in layer-stacking irradiation.

**Supplementary Information:**

The online version contains supplementary material available at 10.1007/s12194-024-00847-1.

## Introduction

Carbon-ion beams have Bragg peak characteristics, thus facilitating the delivery of higher radiation doses to a target and increasing its biological effectiveness. Passive irradiation [1, 2], an irradiation method for particle therapy, employs a constant spread-out Bragg peak (SOBP) width at all locations using ridge filters or range modulators. This feature causes the delivery of an excessive dose to normal tissues, often on the proximal outside of the target. Layer-stacking irradiation resolves this issue by employing a passive irradiation system, forming an irradiation field similar to that of scanning irradiation [3–5]. In layer-stacking irradiation, a mini-peak with an SOBP of several mm is generated. The target volume is divided into layers along the longitudinal direction, and these mini-peaks are shifted to each layer by a range shifter. The irradiation occurs along the beam’s upstream direction, and the irradiation field is adjusted to match the target shape using a multi-leaf collimator (MLC). This method reduces the delivery of excessive doses to organs surrounding the target while maintaining high-dose uniformity within the target. However, if the target moves during irradiation, the dose uniformity inside the target is degraded because of the interplay effect between the layers [6]. Therefore, layer-stacking irradiation is not clinically applied during non-negligible motion.

Tashiro et al. proposed an optimal margin-setting method for respiratory-gated irradiation and smearing techniques to ensure target dose coverage when beam range variations occur during passive irradiation [7]. Several strategies have been proposed for performing three-dimensional (3D) scanning irradiation [8], including the fast rescanning method using respiratory-gated irradiation [9], optimal margins [10], phase-controlled rescanning method [11, 12], and markerless tumor tracking techniques under respiratory gating [13].

Scanning irradiation creates an irradiation field that conforms to the target shape by scanning the target region with a narrow beam produced by an accelerator. Therefore, excessive doses to normal tissues, which remain problematic during passive irradiation, are avoided. However, scanning irradiation with respiratory motion is less robust than passive irradiation [9, 14–16]. In contrast, layer-stacking irradiation is expected to be more robust to motion than scanning irradiation and more conformal than conventional passive irradiation, because each adjusted layer is irradiated simultaneously [17].

Several studies have investigated the dose distribution of layer-stacking irradiation to a moving target [6, 17–19]. In the treatment of lung cancer using four-dimensional (4D) computed tomography (CT), layer-stacking irradiation enables the delivery of higher doses to the target volume compared to passive irradiation while reducing the doses to surrounding normal tissues [17]. Additionally, dose uniformity was reported to be improved when combined with respiratory-gated irradiation [17]. Using a numerical lung phantom, both the dose rate and gating irradiation are reported to enhance dose uniformity [18]. A previous study that conducted a dose uniformity assessment using two-dimensional (2D) physical dose measurements suggested applying additional margins to improve the dose uniformity and coverage of the target [6]. However, applying a common additional margin might result in delivering an excessive dose outside the target, especially when the target motion is minimal. For the interlayer water-equivalent depth variation, a new weight optimization algorithm has also been proposed to improve the stability of depth-dose distributions [19]. Various factors that improve the dose uniformity have been reported; however, the motion tolerance for layer-stacking irradiation has not yet been demonstrated. In clinical practice, the dose uniformity evaluation and acceptable intrafractional target motion amount should be clarified.

Generally, treatment planning systems used in clinical practice cannot simulate the dose distributions for layer-stacking irradiation when considering motion [7]. Therefore, there is a need to develop a dose-calculation system that includes target motion. Additionally, the validity of the simulation must be verified through measurement. Therefore, this study aimed to demonstrate the validity of the simulation by comparing it with the measured physical dose for layer-stacking irradiation with carbon-ion beams. Our goal was to demonstrate a tolerance level for the intrafractional target motion amount by performing clinical dose simulations and evaluating dose uniformity using the validated simulation method.

## Materials and methods

### Target setup

**Fig. 1:**
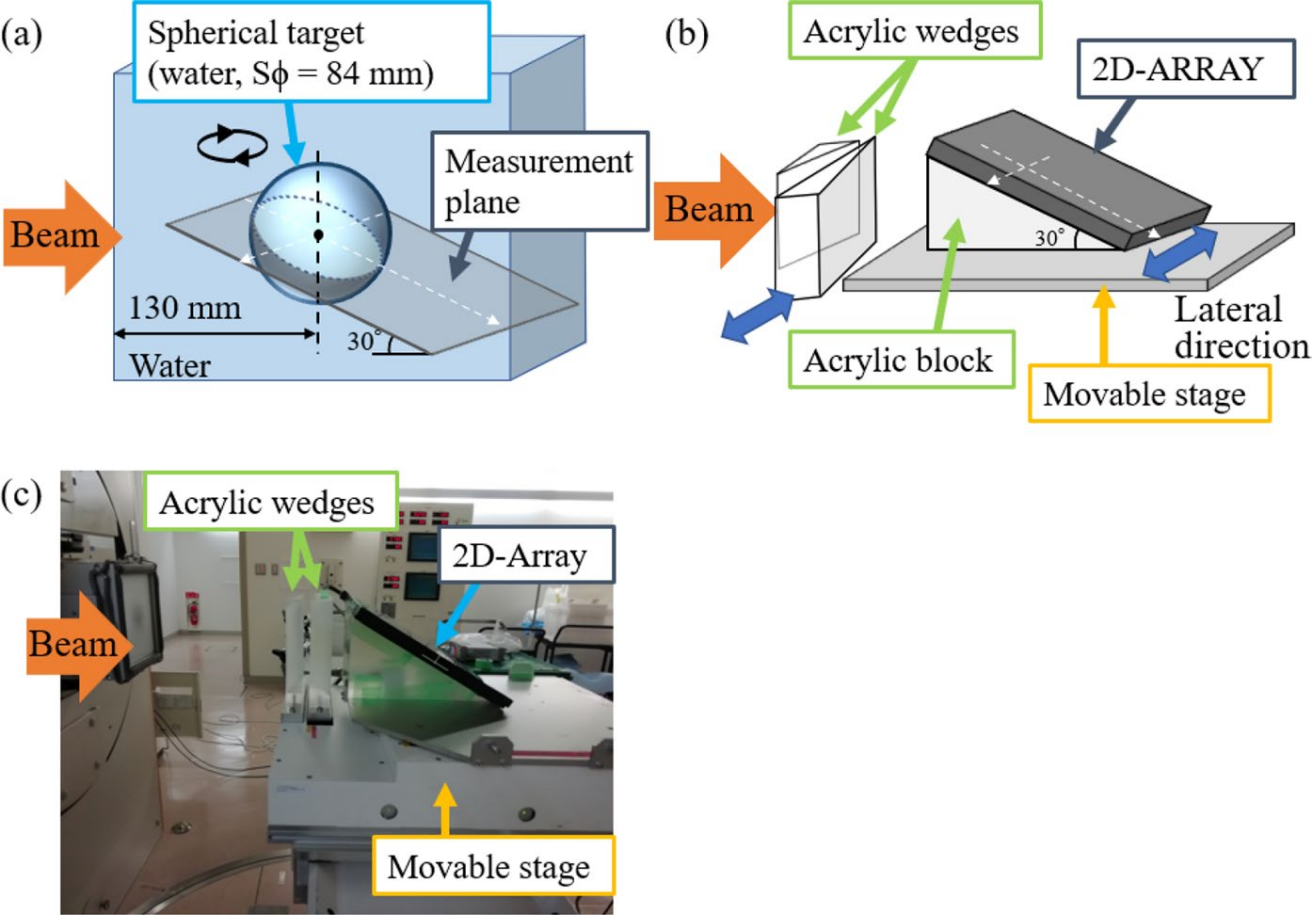
**a** Schematic diagram of the numerical phantom for the spherical target and the measured plane. The target moves in the lateral and/or proximal directions. **b** Schematic and **c** photograph of the measurement setup. The 2D array was positioned to measure the dose through the target center. White arrows in (**a**) and (**b**) correspond to the arrows in Fig. 3a and b

A spherical target with a diameter of 84 mm was placed as the clinical target volume (CTV) in a numerical water phantom at a center depth of 130 mm (Fig. 1a). In this study, a simple water-equivalent system was adopted for the primary purpose of comparison with simulations and measurements, assuming clinical application to bone and soft tissue or liver tumors with densities similar to water in and around tumors. The CTV motion directions were assumed to be in the lateral and/or longitudinal (proximal, beam upstream) to investigate the motion effect. If the CTV motion occurred simultaneously in both directions, an elliptical CTV motion was performed with a phase difference of π/4. The amount of the CTV motion within the planning target volume (PTV) was set to a combination of 0–5, 7, and 20 mm physical length in the lateral direction and 0–5-mm physical length [equivalent to water-equivalent path length (WEPL)] in the proximal direction. Table 1 lists the experimental conditions for the CTV setting and irradiation.

**Table 1:** Experimental conditions for the respiratory motion of the CTV

Diameter/Depth	84 mm/130 mm at the center
Prescribed dose	5 Gy (RBE)
Lateral CTV motion amount during irradiation	0, 1, 2, 3, 4, 5, 7, and 20 mm
Proximal CTV motion amount during irradiation	0, 1, 2, 3, 4, and 5 mm
Respiratory cycle	3.5 s
Phase difference in the motion directions	$\frac{\pi }{4}$
Gating to lateral direction	Yes/No
Gating window	30%

The prescribed dose of 5 Gy (RBE) was used for treatment planning. This determines the prescribed physical dose at the isocenter, which in this case was approximately 2.35 Gy (Fig. 3)

### Treatment planning

The XiO-N treatment planning system (ELEKTA, Stockholm, Kingdom of Sweden and Mitsubishi Electric, Tokyo, Japan) [7] was used to derive plan parameters for both experiments and simulations. The treatment plans in XiO-N were created for PTVs inclusive of internal margins (IM) [7], although they were assumed to be in a static condition. A 3 mm setup margin (SM) [7] was applied in all directions and adjustments were made to beam range and leaf margins to ensure sufficient dose coverage within the PTV in the static condition. For the respiratory-gated irradiation, the IM for a gating window of 30% lateral motion was provided in each direction. In this paper, the CTV motion amount within the gating window is referred to as “CTV motion amount during irradiation”.

### Measurement setup

Irradiation was performed under static and moving conditions described in Sect. 2.1, based on the planned data described in Sect. 2.2. The treatment plans with the prescribed dose of 5 Gy (relative biological effectiveness, RBE) to the PTV were used. The respiratory cycle was set to 3.5 s, offset from the 3 s of the synchrotron operating cycle. The physical dose distributions were measured at least twice for each condition using a cross-calibrated 2D array [OCTAVIUS detector 729XDR (T10031), PTW, Freiburg, Germany] with carbon-ion beams. The plane physical dose distributions were measured by placing the 2D array at a 30-degree angle (Fig. 1b and c). A respiratory motion phantom [KMF-GU-001, Accelerator Engineering Corporation (AEC), Chiba, Japan] was used to simulate the respiratory motion. The lateral CTV motion was performed by shifting the moving stage of the phantom. The longitudinal CTV motion was simulated using a pair of acrylic wedges moving in the lateral direction to cause beam range variation. A laser-type synchronizer, AZ-733VI (Anzai Medical, Tokyo, Japan), was used for respiratory-gated irradiation. The synchronizer was placed to enable the laser to detect the surface of the acrylic block and ensure that the laser was projected in the lateral direction.

### Simulation of 3D physical and clinical dose distributions for layer-stacking irradiation with respiratory motion

In-house C++ software was developed to simulate the 3D physical and clinical dose distributions including the target motion. The simulation utilized the broad-beam method [20, 21] and incorporated simplified wobbling effects and beam scattering, as described later in this section to closely replicate the conditions in XiO-N. The plan parameters, such as MLC, range shifter, range compensator, weighting factor for each layer, tissue–phantom ratio (TPR), and linear-quadratic (LQ) model parameters *α* and *β* for RBE calculations [22], were obtained from the corresponding plan made by XiO-N for the conditions in Table 1.

**Fig. 2:**
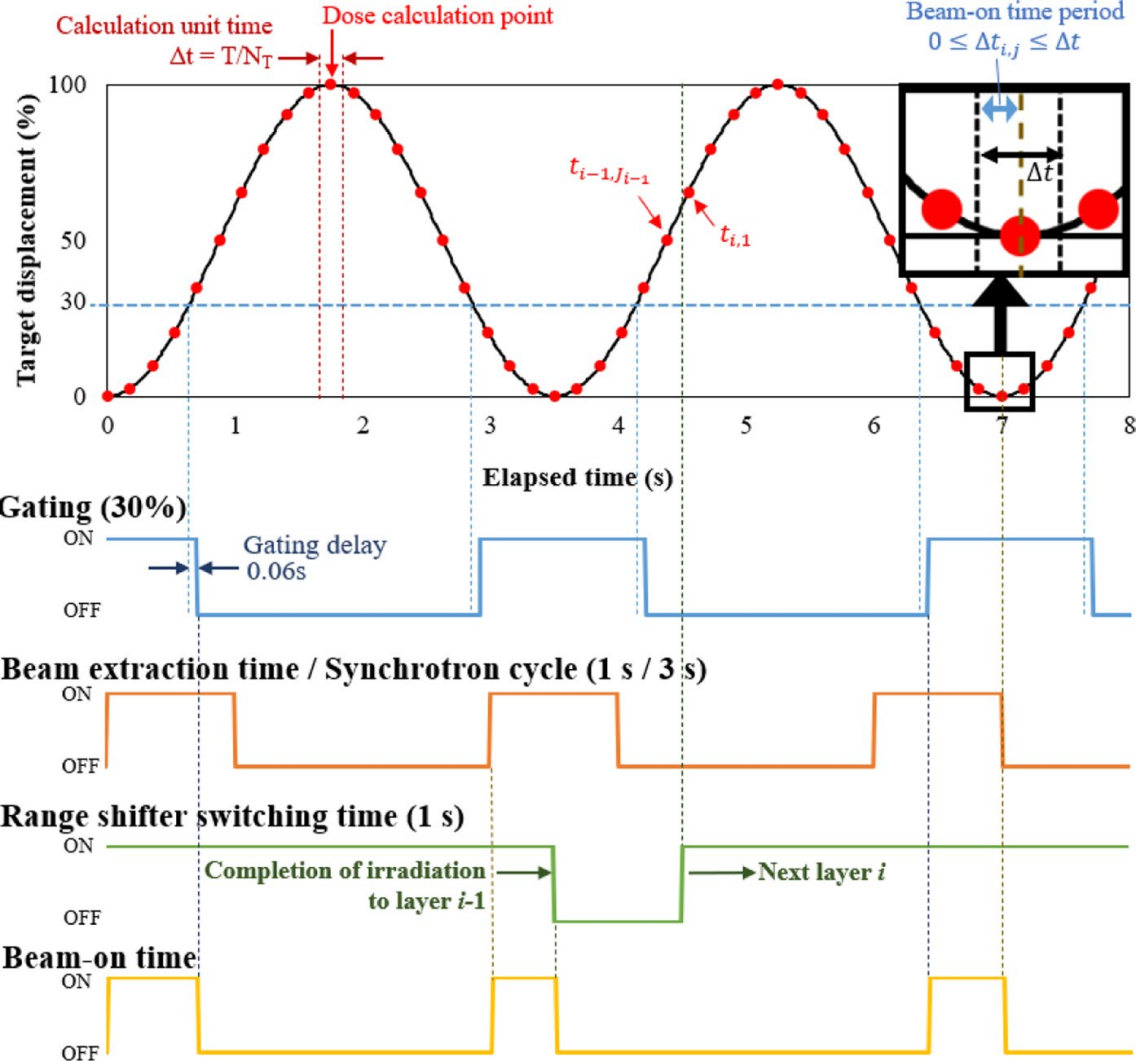
Timing relationship between respiratory motion and irradiation devices

The dose distributions were simulated on a 1-mm grid within a 3D water volume. The parameters of the set values are summarized in Table 2. The temporal relationships between several parameters are shown in Fig. 2. The 3D physical dose distribution $D\left(\textbf{\textit{r}}\right)$ was derived as follows:

1 $$D\left(\textbf{\textit {r}} \right) = \sum_{all \, i}\sum_{all \, j}{\Delta w}_{i, j}d\left({\textbf{\textit {r}}+\Delta \textbf{\textit {r}}}_{i,j}\right),$$

where $i$ was the layer number and $j$ was the elapsed time element within a single layer $i$. $d\left(\textbf{\textit{r}}\right)$ represents the depth-dose distribution for a single layer, obtained from the TPR, the source to axis distance (SAD), and the depth component at position $\boldsymbol{r}$. Due to respiratory motion, the coordinate $\boldsymbol{r}$ receives the dose at the coordinate $\boldsymbol{r}+\Delta \boldsymbol{r}_{i,j}$. The weighting factor ${\Delta w}_{i, j}$ was calculated as follows:

2 $${\Delta w}_{i,j} = \frac{\Delta {t}_{i,j}}{\frac{{D}_{Pres}}{\dot{D}}\times \frac{{T}_{ex}}{{T}_{s}}},$$

where ${D}_{Pres}$ and $\dot{D}$ were prescribed dose and average dose rate throughout the synchrotron cycle, respectively. The beam was extracted at the extraction time ${T}_{ex}$ out of the synchrotron cycle ${T}_{s}$. The denominator in Eq. (2) represents the total time required to irradiate all layers. $\Delta {t}_{i,j}$ represents the beam-on time period within the time interval $\Delta t$ at elapsed time ${t}_{i,j}$ for layer $i$. $\Delta t$ is the calculation unit time interval and is expressed as $\Delta t=T/{N}_{T}$ using the respiratory cycle $T$ and the number of time divisions ${N}_{T}$. In this simulation, we set ${N}_{T}$ = 20. If the beam on/off occurred within the time interval $\Delta t$, the net beam-on time period was derived as $\Delta {t}_{i,j}$. After the completion of irradiation in each layer, the beam was not extracted for 1 s while the range shifter switching. For gated irradiation, the beam was not irradiated during the time period when the CTV was positioned outside the gating window. The weighting factor ${\Delta w}_{i,j}$ for beam-on time period $\Delta {t}_{i,j}$ was accumulated and when it reached a value equal to the single-layer weighting factor ${w}_{i}$, the irradiation of the layer $i$ was completed. The relationship between ${w}_{i}$ and ${\Delta w}_{i,j}$ was given by the following equation:

**Table 2:** Simulation conditions for the respiratory motion of the target

CTV	Original CTV^*a^ or CTV_V_^*b^
Prescribed dose	4, 5, 10 Gy (RBE) to PTV
Dose rate	5 Gy (RBE)/min
Lateral CTV motion amount during irradiation	0–20 mm
Proximal CTV motion amount during irradiation	0–20 mm
Respiratory cycle	2–10 s
Beam extraction time (${T}_{ex}$)/synchrotron cycle (${T}_{s}$)	1.0 s/3.0 s
Range shifter switching time	1.0 s
Gating	Yes/No
Gating level	30%
Gating delay time	0.06 s
Number of divisions for respiratory cycle (${N}_{T}$)	20
Phase difference between the respiratory motion and synchrotron cycle (${\theta }_{0}$)	0, $\frac{\pi }{7}$, $\frac{\pi }{5}$, $\frac{\pi }{3}$, $\frac{\pi }{2}$, $\pi$,$\frac{3\pi }{2}$

Table 1 is the original conditions and additional condition for simulation is listed in Table 2

^*a^ “Original CTV”: the CTV in the measurement condition (Table 1)

^*b^ “CTV_V_”: variable CTV; the condition under which the CTV was created by inversely subtracting IMs from the fixed PTV, with 20 mm lateral and 5-mm proximal margins to the original CTV

3 $${w}_{i}= \sum_{all\,j}{\Delta w}_{i,j}.$$

Assuming a horizontal beam (gantry angle = 90°) and defining the 3D coordinates as ${\boldsymbol{r}}=\left(x,y,z\right)=$ (posterior-anterior, lateral (inferior-superior), depth (left–right)) direction, the coordinate ${\boldsymbol{r}+\Delta\boldsymbol{r}}_{i,j}$ and its respective elements were determined as follows:

4 $${\boldsymbol{r}+\Delta\boldsymbol{r}}_{i,j}={\left(x+{\Delta x}_{i,j}, {y+\Delta y}_{i,j},{z+\Delta z}_{i,j}\right)},$$

5 $$\Delta {x}_{i,j}=0,$$

6 $$\Delta {y}_{i,j}={L}_{i,j},$$

7 $${\Delta z}_{i,j}={P}_{i,j}+{S}_{i}+B\left(x+{\Delta x}_{i,j}, {y+\Delta y}_{i,j}\right).$$

Range shifter thickness was calculated as ${S}_{i}={S}_{1}+\left(i-1\right)\times 2.5$ mm-WEPL and inserted after the end of irradiation in each layer to adjust the beam range. ${S}_{1}$ is the initial thickness at $i$ = 1. $B\left(x, \text{ y}\right)$ is the thickness of the range compensator (bolus) in the coordinates of the beam vertical plane. ${L}_{i,j}$ and ${P}_{i,j}$ are the lateral and longitudinal amounts of the respiratory motion relative to the elapsed time, expressed as follows:

8 $${L}_{i,j}=\frac{{A}_{L}}{2}\left\{1-\text{cos}\left(\frac{2\pi {t}_{i,j}}{T}+{\theta }_{0}\right)\right\},$$

9 $${P}_{i,j}=\frac{{A}_{P}}{2}\left\{1-\text{cos}\left(\frac{2\pi {t}_{i,j}}{T}+\frac{\pi }{4}+{\theta }_{0}\right)\right\},$$

where ${A}_{L}$ and ${A}_{P}$ are the amplitudes from the maximum exhalation to the maximum inhalation. When the CTV moved in two directions simultaneously, the phase difference in the proximal direction relative to the lateral direction was set to $\pi /4$. ${\theta }_{0}$ is the initial phase of the respiratory motion with respect to the synchrotron cycle, and seven patterns were verified. The initial phase changes caused a shift in the timing of beam irradiation. The elapsed time ${t}_{i,j}$ represents the dose-calculation time point set at the center of the time interval $\Delta t$

10 $${t}_{i,j} = {t}_{i-1,{J}_{i-1}}+j\Delta t,$$

where ${J}_{i}$ is the final time element at the layer number $i$. We defined ${t}_{\text{0,0}} = -\Delta t$, because the starting time point was set as ${t}_{\text{1,1}} = 0$.

The RBE and clinical dose $C\left(\boldsymbol{r}\right)$ were calculated as follows, based on the formulated clinical RBE [4] and the range-modulated beam method [22]:

11 $$RBE{\boldsymbol{(r)}}= \frac{1.46\times 4.035\times 2{\beta }_{mix}\left(\boldsymbol{r}\right)}{\sqrt{{{\alpha }_{mix}\left(\boldsymbol{r}\right)}^{2}-4{\beta }_{mix}\left(\boldsymbol{r}\right)\text{ln}0.1}-{\alpha }_{mix}\left(\boldsymbol{r}\right)},$$

12 $$C\left(\boldsymbol{r}\right) = RBE\left(\boldsymbol{r}\right)D\left(\boldsymbol{r}\right).$$

The clinical factor 1.46 employed in the Gunma University Heavy Ion Medical Center (GHMC) was used. ${\alpha }_{mix}\left(\boldsymbol{r}\right)$ and ${\beta }_{mix}\left(\boldsymbol{r}\right)$ are the coefficients of the LQ model for the mixed beam obtained as dose averages including the motion as follows:

13 $${\alpha }_{mix}\left(\boldsymbol{r}\right)= \sum_{all \, i}\sum_{all\, j}f\left({{\boldsymbol{r}}+\Delta {\boldsymbol{r}}}_{i,j}\right)\alpha \left({{\boldsymbol{r}}+\Delta {\boldsymbol{r}}}_{i,j}\right),$$

14 $${\sqrt{{\beta }_{mix}\left(\boldsymbol{r}\right)}= \sum_{all \, i}\sum_{all \, j}f\left({\boldsymbol{r}+\Delta \boldsymbol{r}}_{i,j}\right)\sqrt{\beta \left({\boldsymbol{r}+\Delta \boldsymbol{r}}_{i,j}\right)}},$$

where $\alpha (z)$ and $\beta (z)$ are coefficients of the LQ model obtained from XiO-N corresponding to the depth-dose distribution $d(z)$ in a single layer. $f\left({\boldsymbol{r}+\Delta \boldsymbol{r}}_{i,j}\right)$ is given by the following equation:

15 $$f\left({\boldsymbol{r}+\Delta \boldsymbol{r}}_{i,j}\right)= \frac{\Delta {w}_{i, j}d\left(\boldsymbol{r+\Delta \boldsymbol{r}}_{i,j}\right)}{D\left(\boldsymbol{r}\right)}.$$

Parallel beams were used to simplify the simulations due to the fact that the SAD was approximately 7 m, which was considered sufficiently long. An approximated factor that accounts for the slight increase in the lateral dose distribution near the wobbling radius was employed and applied at all depths. The factor was defined as the ratio of the planned value of the lateral dose distribution by XiO-N to the simulated one before the wobbling correction at the isocenter depth. To simply account for multiple-beam scattering, the lateral dose distributions were convoluted using a Gaussian distribution with a standard deviation (SD) of 3.8 mm, which was determined to best fit the planned and simulated physical dose distributions on the isocenter plane. This convolution was carried out for the simulated physical and clinical dose distributions. For static conditions with combinations of the lateral and longitudinal IMs shown in Table 1, average values of gamma passing rates (3%/3 mm) and SDs for the physical and clinical dose distributions between XiO-N and the simulation were 99.94 ± 0.04% and 99.91 ± 0.02%, respectively. The simulation reproduced the dose distribution in XiO-N in static conditions.

Gating was employed at 30% amplitude when the target moved in a single direction. When the CTV moved in two directions simultaneously, the beam was gated with respect to the lateral motion. The gating delay time between the actual motion and the gating signal was set to 0.06 s, which was used in clinical practice.

### Comparison of measured and simulated physical dose distributions

For comparison with measurements, the physical dose distributions were simulated with the setting of the prescribed dose of 5 Gy (RBE). Two methods were used to evaluate the agreement between the measured and simulated physical dose distributions.

First, a gamma analysis [23] was conducted. The criterion was set to 3%/3 mm, considering the sensitive volume of the detectors and experimental setup errors. The γ value with the best agreement at each chamber position was obtained between the average of the measured physical dose distributions and the simulated ones for the various initial phases.

Second, dose coverage inside the CTV was compared between measurement and simulation. The physical doses were not constant in the SOBP for the uniformly prescribed clinical dose. Therefore, the dose variation from the static condition was examined to evaluate the dose coverage for the CTV motion. The simulated physical dose distributions corresponding to the area measured by the 2D array were extracted. The averaged physical doses on the incident surface of the sensitive volume in each ionization chamber were obtained. Finally, the delta 2D conformity index (ΔCI_2D_), defined as the ratio of the number of ionization chambers within a ± 5% dose difference in the CTV area, was estimated.

### Clinical dose evaluation

The clinical dose distributions were simulated at 4 and 10 Gy (RBE). The respiratory cycle was examined within a range of 2–10 s to investigate the effect on dose uniformity. Additionally, to examine more motion conditions, we defined the variable CTV (CTV_V_) created by inversely subtracting IMs from the fixed PTV [6], with 20-mm lateral and 5-mm proximal margins to the original CTV presented in Table 1. As a result, the CTV_V_ was adjusted according to changes in the CTV_V_ motion amounts. The CTV_V_ offered the advantage of avoiding the necessity to create individual treatment plans for various respiratory motion, because the single fixed PTV was used.

The acceptable target motion amounts during irradiation were derived from the evaluation of the clinical dose uniformity. The conformity index (CI; defined as (V_95_ − V_105_)/CTV), was employed, because the CTV dose coverage was important when considering motion. The differences in CI values between the motion condition and the static condition, denoted as ΔCI (= CI_move_ − CI_static_), were obtained to remove the effect of differences in the CI values in the static conditions. When the average value for all initial phases was within ± 5%, it was deemed clinically acceptable. The worst averaged ΔCI value for various respiratory cycles was defined as ΔCI_worst_. The maximum ΔCI_worst_ value within ± 5% for motion conditions was regarded as ‘acceptable CTV motion amount during irradiation’. In the case of gating, this refers to the target motion amount within gating window.

## Results

### Measured vs. simulated physical dose distributions

**Fig. 3:**
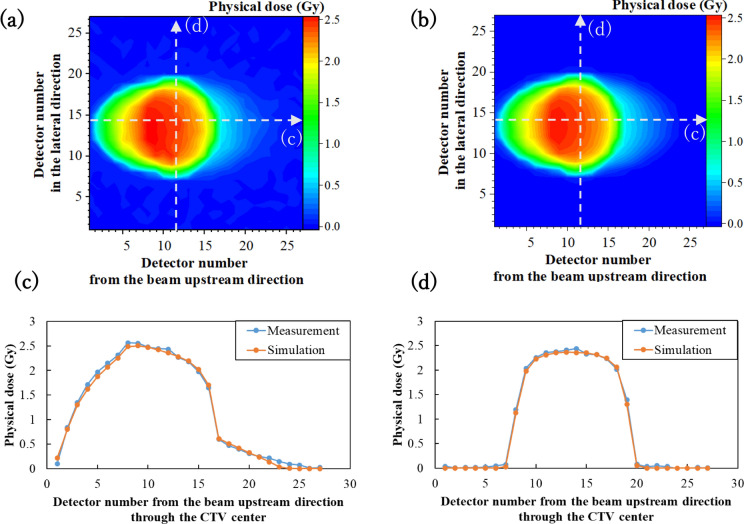
Top: **a** Measured and **b** simulated 2D physical dose distributions for the static CTV with margins of (Lateral, Proximal) = (7, 0). Bottom: **c** and **d** Measured and simulated dose profiles along the axes indicated by the white dashed arrows in (**a**) and (**b**). Corresponding arrows are indicated in Fig. 1a and b

**Fig. 4:**
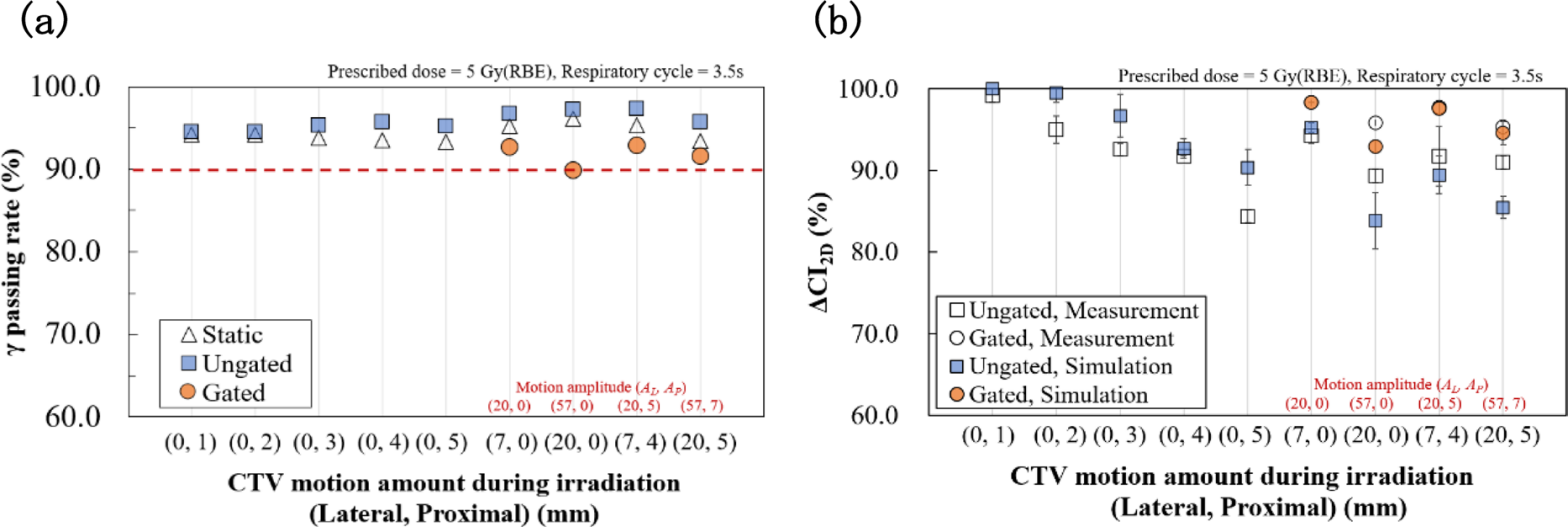
**a** Gamma analysis with the gamma passing rate and **b** ΔCI_2D_ for the CTV motion amount during irradiation in lateral and proximal directions. e.g. (Lateral, Proximal) = (0, 1). The criterion is 3%/3 mm. Static condition refers to the irradiation of the static target based on the respective PTV. Red letters on the horizontal axis represent the CTV motion amplitude (*A*_*L*_, *A*_*P*_) in case of respiratory-gated irradiation

Figure 3 shows examples of the measured and simulated physical dose distributions for the static CTV with a 7 mm lateral margin. The measured and simulated dose distributions were nearly consistent. Figure 4a shows the gamma passing rate for a combination of CTV motion in the lateral and longitudinal directions. The gamma passing rates almost exceeded 90%, indicating that the measured and simulated dose distributions matched. The results confirm the validity of the simulation.

Figure 4b shows the simulated and measured ΔCI_2D_ results. The measured SDs (error bars) were due to the number of measurements, while the simulated SDs were due to the initial phase differences for the CTV motion. ΔCI_2D_ values almost worsened with the increasing motion amount of the target. Respiratory-gated irradiation improved ΔCI_2D_ values for the same CTV motion during irradiation [e.g., (7, 0) ungated and (7, 0) gated]. Gated irradiation also improved ΔCI_2D_ values for the same respiratory motion amplitudes [e.g., (20, 0) ungated and (7, 0) gated]. Overall, a concordance was observed between the measurement and simulation.

### Clinical dose uniformity assessment and acceptable CTV motion

**Fig. 5:**
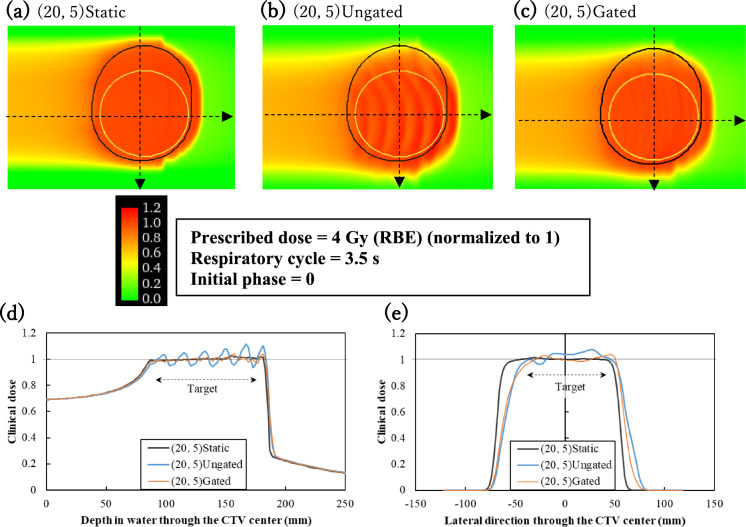
Simulated clinical dose distributions for PTV with margins of 20 mm in the lateral and 5 mm in the proximal directions, corresponding to the CTV motion (20, 5). The dose is normalized from the prescribed dose of 4 Gy (RBE) to 1. Top: Two-dimensional clinical dose distributions for the static (**a**) and moving [un-gating (**b**) and gating (**c**)] targets. Bottom: Depth (**d**) and lateral (**e**) dose distributions for the static and moving targets. The yellow and black contours represent the CTV and PTV, respectively. The calculated axes are indicated as the dashed arrows in (**a**), (**b**), and (**c**)

Figure 5 shows examples of the simulated lateral–depth 2D clinical dose distributions for static (a) and moving [un-gating (b) and gating (c)] CTV through the CTV center. The CTV motion along the longitudinal direction caused rippling, hot spots, and cold spots in the dose distribution in the ungated condition (d). Notably, for the (20, 5) gated, the motion amplitude was (57, 7), and the dose distribution was less disturbed than that for (20, 5) ungated. In the lateral dose distribution (e), the dose shifted because of the lateral CTV motion in both un-gating and gating conditions. However, dose uniformity within the target region was ensured with gating.

**Fig. 6:**
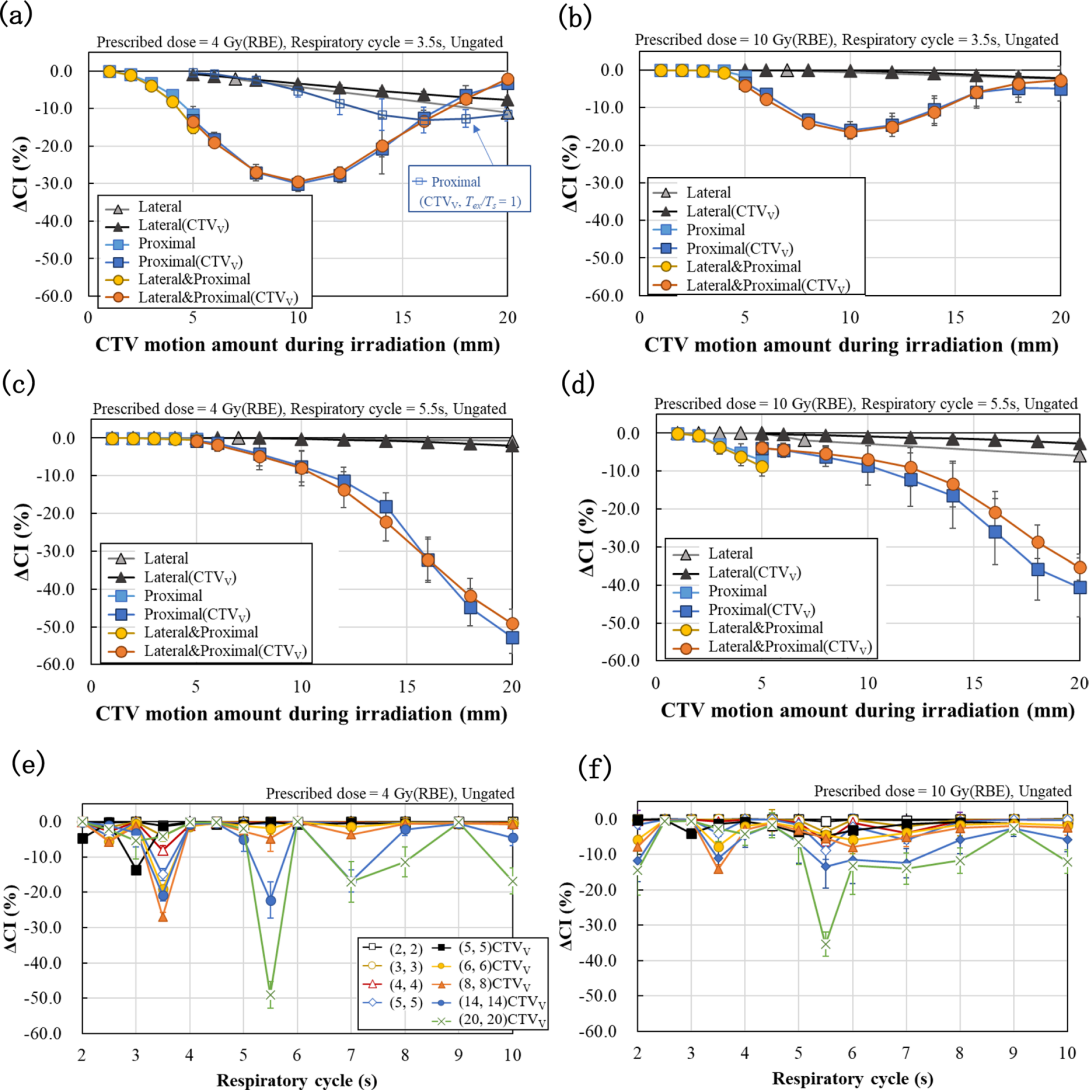
ΔCI for the CTV motion amount during irradiation (**a**)–(**d**) or respiratory cycles (**e**) and (**f**) in the lateral and/or longitudinal directions for the prescribed doses of 4 and 10 Gy (RBE) under the ungated condition. “Lateral and Proximal” indicate the CTV motion in both lateral and longitudinal directions, e.g., (4 mm, 4 mm). Proximal (CTV_V_, *T*_*ex*_*/T*_*s*_ = 1) in (**a**) indicates the condition when the beam is continuously extracted regardless of the synchrotron cycle

Figure 6 shows ΔCI values for the CTV motion amount during irradiation (a)–(d) or respiratory cycle (e) and (f) under ungated condition at 4 or 10 Gy (RBE). The SDs were due to the initial phase differences. Including the lateral direction (a)–(d) or respiratory cycle of 5.5 s (c) and (d), ΔCI values degraded with increasing CTV motion amount (a)–(d). For CTV motion in the longitudinal direction with a respiratory cycle of 3.5 s (a) and (b), ΔCI values initially decreased and then improved when involving CTV motion in the proximal direction.

Figure 6e and f show ΔCI values for respiratory cycles under ungated condition. ΔCI values for respiratory cycles of 3.5 and 5.5 s in Fig. 6e and f correspond to those for the lateral and proximal conditions in Fig. 6a–d. The respiratory cycle dependences of ΔCI including motion amount with ungated conditions in the lateral or proximal direction at 4 and 10 Gy (RBE) are shown in Supplementary Fig. 1. When including the longitudinal motion [Supplementary Figs. 1c–f], ΔCI values largely worsened across several respiratory cycles. The motion condition that resulted in the worst ΔCI value varied depending on the respiratory cycle. To investigate the effect of beam extraction timing, dose distributions were evaluated under several conditions with *T*_*ex*_*/T*_*s*_ = 1, which meant that the beam was continuously extracted regardless of the synchrotron operating cycle (Fig. 6a). ΔCI values largely improved compared to the case where the synchrotron cycle and beam extraction timing were considered [proximal (CTV_V_) in Fig. 6a].

Supplementary Figure 2 shows ΔCI values with gating for respiratory cycles at 4 and 10 Gy (RBE). For single motion directions [Supplementary Figs. 2a–d], a smaller prescribed dose resulted in a greater variation of ΔCI values across respiratory cycles. ΔCI values were more affected in the longitudinal motion than in the lateral motion. For lateral and longitudinal motion [Supplementary Figs. 2e and f], ΔCI values almost showed greater variation at 4 Gy (RBE), while ΔCI values degradation were more pronounced at 10 Gy (RBE) than 4 Gy (RBE) when the motion amounts of the target were greater than 14 mm.

Supplementary Figure 3 compares ΔCI values versus respiratory cycle for the gated and ungated irradiations for each motion condition at 4 and 10 Gy (RBE). At the same CTV motion amount during irradiation, ΔCI values almost improved for some respiratory cycles by gated irradiation. When comparing cases where the CTV motion amplitudes were nearly identical [e.g., (7, 0) Gated in Supplementary Fig. 3a and b vs. (20, 0) Ungated in Supplementary Figs. 3e and f], ΔCI values generally showed improvement and stability across most respiratory cycles with gating.

**Fig. 7:**
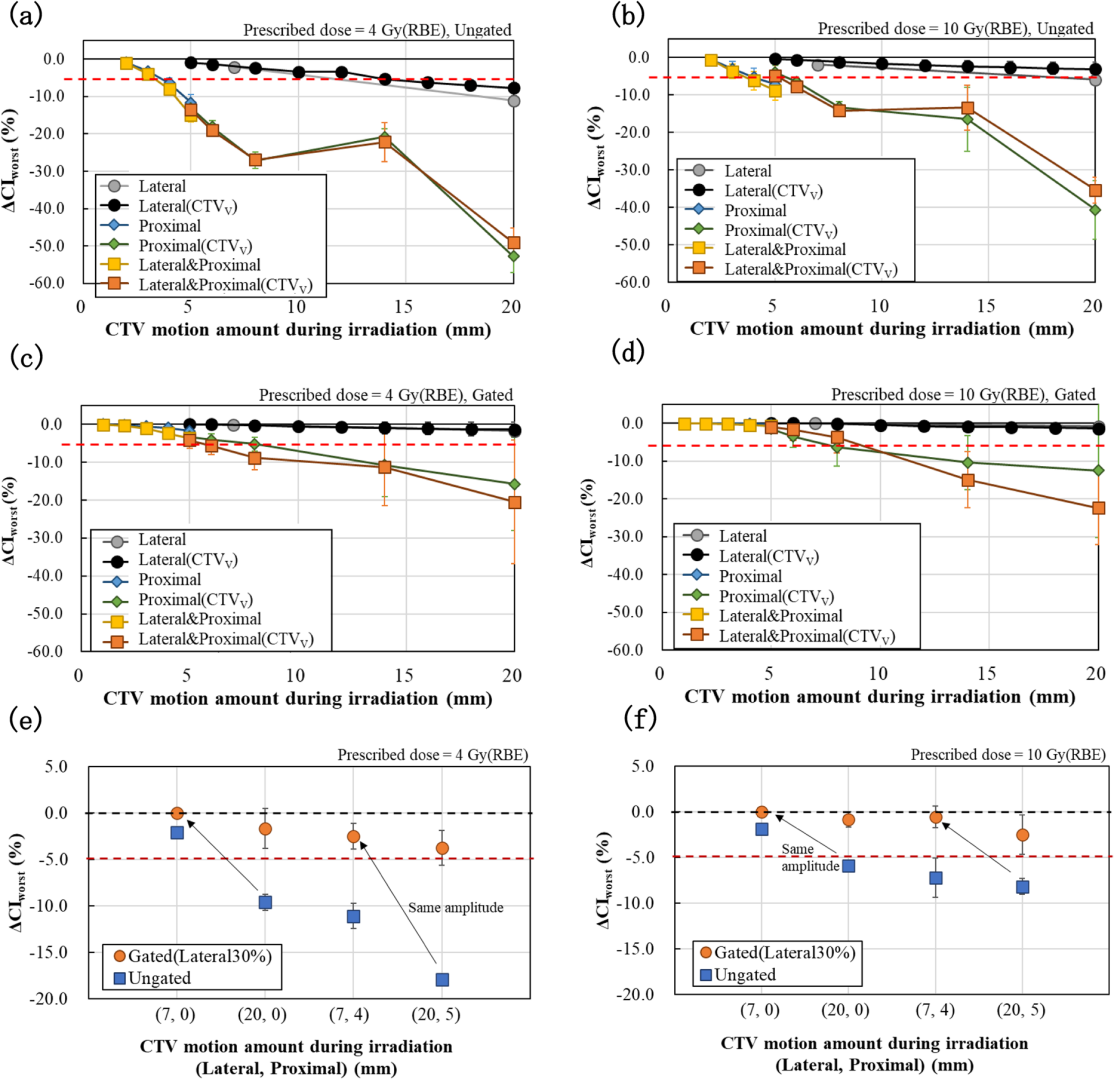
ΔCI_worst_ for motion amounts of the target during irradiation at prescribed doses of 4 and 10 Gy (RBE) in the ungated (**a**) and (**b**)/gated (**c**) and (**d**) condition. **e** and **f** Conditions with the same motion amount during irradiation (horizontal axis) and motion amplitude (black arrows) can be compared simultaneously. For all motion conditions in this figure, trends in ΔCI values for each respiratory cycle are shown in Supplementary Figs. 1–3

Figure 7 shows ΔCI_worst_ for the motion amount during irradiation. ΔCI_worst_ values showed a monotonous degradation with respect to almost all CTV motion amounts during irradiation. Slight differences in ΔCI values between CTV and CTV_V_ were observed at ungated 20 mm in the lateral direction and ungated 5 mm including the longitudinal motion. Outside of these conditions, however, little difference was observed. ΔCI_worst_ values also worsened with lower prescribed doses. Comparing ungated and gated conditions (Fig. 7a and b vs. c and d or Fig. 7e and f), ΔCI_worst_ values overall improved under gated conditions for the same motion amounts during irradiation. The arrows in Fig. 7e and f indicate the comparison from un-gating to gating conditions for the same motion amplitude of the target. When the lateral motion amplitude was 20 mm and gated with a 30% gating window, the motion amount during irradiation was approximately 7 mm (black arrow). ΔCI_worst_ values (Fig. 7e and f) also improved with gated irradiation for the same CTV motion amplitude.

Table 3 summarizes the acceptable CTV motion amounts during irradiation from the maximum motion amounts among the accepted ΔCI_worst_ values in Fig. 7. Based on the concept of employing worst-case values in this study, the worse ΔCI_worst_ values between CTV and CTV_V_ were adopted to derive the acceptable motion amounts. In the ungated condition, ΔCI_worst_ values for the original CTV in the lateral direction were slightly worse than those for the CTV_V_ condition in Fig. 7a and b. From the linear interpolation between (7, 0) ungated and (20, 0) ungated conditions, acceptable target motion amounts were derived. The acceptable motion amounts during irradiation increased with higher prescribed doses or gated irradiation. When including proximal motion, acceptable CTV motion amounts varied 0–2 mm and were not greatly affected by lateral motion. The acceptable motion amount during irradiation in the lateral direction was greater than in the longitudinal direction. They also increased by at least 6 mm with an increase in the prescribed dose and with gating. Including longitudinal directional motion and ungated condition, the acceptable motion amount was around 3 mm, regardless of one or two directions. When gating was applied, the acceptable motion amount increased by up to 5 mm.

**Table 3:** Acceptable CTV motion amount (mm) during irradiation determined from ΔCI_worst_

	4 Gy (RBE)		10 Gy (RBE)	
	Lateral	Proximal	Lateral	Proximal
Ungated (single direction)	11	3	17	4
Gated (single direction)	≥ 20	6	≥ 20	6
Ungated (combination)	3		3	
Gated (combination)	5		8	

In gated (single direction) condition, CTV motion amount during irradiation indicates when 30% of the amplitude for each direction is gated. Combination condition indicates the case of motion amounts in both lateral and proximal direction at the same time, e.g., (Lateral, Proximal) = (3, 3). Gated (combination) is the condition when 30% of the lateral amplitude is gated

## Discussion

In this study, physical dose distributions were both measured and simulated, and the results were compared to demonstrate the validity of the simulated dose distributions for layer-stacking irradiation. This study is the first to suggest a metric for acceptable target motion amounts, which is based on a clinical dose uniformity assessment under various conditions, encompassing different respiratory cycles, motion amounts, prescribed doses, initial phase for respiratory motion, and gating/non-gating. Furthermore, dose distribution simulations including respiratory motion were performed at fine time intervals to reflect more realistic situations such as gating delay, beam extraction time, synchrotron cycle, range shifter switching time, and initial phase.

The gamma passing rate (Fig. 4a) and ΔCI_2D_ (Fig. 4b) evaluations as well as the comparison of dose distributions shown in Fig. 3 support the validity of the dose distribution simulations. The measurement produced minute noise. Notably, the gamma passing rates were lower with gating (Fig. 4a), possibly owing to the approximately threefold longer irradiation time and slight enhancement of noise. Another reason for the difference between measurements and simulations may be that the simulation does not fully reproduce the actual beamline and experimental setup, and the intrinsic difference may be included in the dose-calculation algorithm. Further investigation into lower gamma values will be needed. However, the validity of the simulation is confirmed considering that the measured and simulated dose distributions were in good agreement, as shown in Fig. 3. The difference between the dose distribution under static and moving conditions (Fig. 4b) shows that the dose uniformity improved with gating in both simulations and measurements. ΔCI_2D_ values (Fig. 4b) displayed a similar trend, with minor differences between measured and simulated dose distributions. The total number of ionization chambers within the CTV was 61. If the dose difference exceeded ± 5% at one chamber, the ΔCI_2D_ degraded by 1.64%. Therefore, a slight dose variation may result in a large change in the ΔCI_2D_ value.

For the lateral motion of the target and ungated condition, ΔCI and ΔCI_worst_ values worsened monotonically with increasing target motion [Supplementary Figs. 1a and b]. Lateral target motion near the target center was covered as PTV, ensuring dose uniformity. On the other hand, the range variation due to the bolus shape was greater near the lateral edge of the target, which can deteriorate dose uniformity. However, these effects are not major, and thus, the impact of beam extraction timing and initial phase for respiratory motion may hardly be observed.

Including longitudinal motion, ΔCI values varied greatly for changes in the target motion amounts, respiratory cycles, and prescribed doses compared to lateral directional motion, as shown in Fig. 6 and Supplementary Figs. 1–3. The changes in dose distribution induced by longitudinal motion differ from those induced by lateral motion in terms of layer overlap. The relationship between the respiratory cycle and the beam extraction timing may change the dose profiles and overlap positions of the layers, which may have a major impact on accumulated dose distribution. Including longitudinal motion and ungated irradiation, ΔCI_worst_ values improved for decreasing target motion and increasing prescribed dose (Fig. 7a and b). This trend is similar to the results of clinical dose assessment using lung numerical phantoms reported by Mori et al. [18]. On the other hand, ΔCI values did not always show a monotonous increasing or decreasing trend with target motion, respiratory cycle, and prescribed dose, as shown in Fig. 6 and Supplementary Figs. 1–3. This is thought to be a result of changes in irradiation timing due to the beam extraction time/synchrotron cycle. One of the major differences from the work by Mori et al. is the consideration of the synchrotron operating cycle. The simulated ΔCI values in the case of continuous beam irradiation except for the range shifter switching time for the proximal (CTV_V_, *T*_*ex*_*/T*_*s*_ = 1) in Fig. 6a were substantially better than those when the beam is irradiated for 1 s during the 3 s synchrotron operating cycle (*T*_*ex*_*/T*_*s*_ = 1/3). As a result, an almost monotonic change in ΔCI values was obtained. In the case of *T*_*ex*_*/T*_*s*_ = 1, the flatter accumulated SOBP was achieved due to the continuous irradiation during each layer. On the other hand, dose uniformity may be improved depending on the combination of irradiation timing, target motion amount and respiratory cycle [e.g., proximal (CTV_V_) 20 mm in Fig. 6a]. The synchrotron operating cycle has a large influence on the dose distribution and was therefore important for a more realistic assessment of the dose distributions.

When the target motion amplitude was the same, respiratory-gated irradiation improved ΔCI and ΔCI_worst_ values and reduced variability due to differences in respiratory cycle, as shown in Figs. 7e and f, and gated conditions in Supplementary Fig. 3a, b, e, and f vs. ungated condition in Supplementary Fig. 3c, d, g, and h. This may be caused by a reduction in the motion amount during irradiation by gating. Comparing the same motion amounts during irradiation, ΔCI values mostly improved by gating as shown in Fig. 7e and f and as shown by the gated vs. ungated conditions in Supplementary Fig. 3. This may be due to differences in the motion probability distribution of the target during irradiation [7], that is, the spatial probability distribution of the target is monomodal or bimodal under gated or ungated conditions, respectively.

In gated irradiation, the beam-on timing was affected by the gated timing in addition to changes in the irradiation timing due to the beam extraction time/synchrotron cycle. Therefore, the trend of ΔCI due to the respiratory cycle was considered to be different from that in ungated condition (Supplementary Figs. 1–3). This also applies to changes in prescribed doses. Changes in the prescribed dose cause variations in the irradiation time during one layer, altering the irradiation timing and having impact on dose uniformity. However, as seen in ΔCI_worst_ (Fig. 7), larger target motion and smaller prescribed dose indicate worse ΔCI_worst_. Trends in ΔCI_worst_ values agree with the previous works [6, 17, 18].

In ungated case, changes in the initial phase ${\theta }_{0}$ had a small effect on the variation in dose uniformity. The maximum SDs for all conditions in Supplementary Fig. 1 was 9.58%. On the other hand, the maximum SD for the gated conditions was 17.73% (Supplementary Fig. 2), approximately twice as high as in un-gating. The variations in SD were particularly large under conditions involving the motion in the longitudinal direction more than 14 mm. This may also be considered by the complexities of beam-on timing due to the synchrotron operating cycle and gating. In Supplementary Fig. 2f, (20, 20) CTV_V_ with a respiratory cycle of 4 s, the ΔCI value was −0.6% in one initial phase and around −30% in other initial phases, resulting in a high SD variability. High dose-uniformity may be obtained by chance when only one irradiation timing is employed. Moreover, as the actual irradiation timing is uncontrolled and random, the assessments of dose distribution at several initial phases are considered necessary.

In “Proximal” and “Lateral and Proximal” conditions with gating (Fig. 7c and d), the gating levels in the longitudinal direction were, respectively, 30% and about 70%, the latter due to lateral gating. Nevertheless, both ΔCI_worst_ values were almost identical up to an amount of motion within the 5% tolerance. Therefore, it is important to set the gating level, so that the target motion amounts during irradiation are within acceptable limits, regardless of the gating level, to maintain dose uniformity.

ΔCI values for CTV and CTV_V_ with the same motion amounts can be used to verify the effect of the target volume variations. In the 5-mm Lateral and Proximal condition in Fig. 7b, there is approximately a 4% difference in averaged ΔCI_worst_ values between CTV_V_ and CTV. For example, in the 5-mm Lateral and Proximal condition of Supplementary Fig. 1f, the mean difference in average ΔCI_worst_ between the original CTV (309 cm^3^) and CTV_V_ (391 cm^3^) for all respiratory cycles was smaller, 1.40%. Furthermore, when the other prescribed dose and gating were performed (Supplementary Fig. 1c-f and Supplementary Fig. 2c-f), there was hardly any difference in the mean ΔCI between the two. The volume changes did not have a major effect on dose uniformity within the range of volume changes examined in this study. Near the motion tolerance, volume changes may have affected the acceptable amount of target motion. We adopted the worst ΔCI value for respiratory cycle, although actual respiratory cycle of patients is not expected to be exactly constant [11]. The respiratory cycle dependence for dose uniformity is predicted to be blurred in actual patients; thus, taking the average of ΔCI values for each respiratory cycle may be practical.

The acceptable target motion amounts during irradiation were determined for the prescribed dose and direction of motion with and without gating (Table 3). The target motion in the longitudinal direction was considered as a change in irradiation depth. For example, in the context of liver application, our study addresses variations in beam range caused by diaphragm motion. In GHMC, irradiation was not performed with 2 mm or more of motion, whereas this study found that 3 mm of motion in the longitudinal direction was acceptable at 4 Gy (RBE). Since gated irradiation required approximately three times the treatment time in this study, investigating the conditions under which ungated irradiation is acceptable would be beneficial in terms of reducing irradiation time and patient burden. Additionally, it may be useful to explore the upper limits of motion with gating.

In dose uniformity evaluations used for deriving acceptable motion amount, it is difficult to measure 3D physical dose distribution. However, comparison of the simulated ΔCI with the measured ΔCI_2D_ might assist in evaluating the reliability of target dose uniformity. ΔCI_2D_ in Fig. 4 and ΔCI in Fig. 6a, Supplementary Figs. 3a, c, e, and g are almost identical conditions and exhibit the same trend. These conditions with a 3.5-s respiratory cycle had the worst ΔCI for various respiratory cycles and were identical to ΔCI_worst_ (Fig. 7), especially in the ungated conditions. These conditions also include motion close to the tolerance range of 1–5 mm in the proximal direction. We consider these results to be complementary to ΔCI and acceptable motion amount, although the measured ΔCI_2D_ was limited to a 2D plane and insufficient for evaluation of 3D dose uniformity.

This study has several limitations. While we used a water phantom considering the simplicity of the system, it may be useful for application to the liver and bone and soft tissue. Therefore, further validation is needed for heterogeneous systems, including lung cancers. Given the potential for unexpected range variations caused by the target motion in the lateral direction, stemming from internal inhomogeneities, it would be safer to adopt an acceptable CTV motion amount in the longitudinal direction rather than in the lateral direction. In clinical practice, single-directional motion is uncommon, and range variation is caused by the lateral motion as well. Therefore, it is important to evaluate variations in water-equivalent depth to the target with respiratory motion using 4D-CT. The tolerance of ΔCI_worst_ <  ± 5% employed in this study may be changed depending on a case-by-case and facility-by-facility basis. To apply the acceptable motion amount derived in this study to actual patients, it is necessary to measure multiple planes in the quality assurance (QA) plan with a motion phantom. Future work should include dose distribution evaluation using heterogeneous phantoms and 4D-CT data from actual patients to examine the relevance of the present metrics.

## Conclusion

In this study, clinical dose uniformity was assessed, and the acceptable target motion amount during irradiation was determined using validated simulations. In both the lateral and longitudinal directions, motion amounts of 3 mm for non-gating and 5 mm for gating were deemed acceptable. The acceptable motion amounts increased by 1–9 mm with higher prescribed dose or when employing of respiratory-gated irradiation. These results employed worst-case scenarios considering multiple respiratory cycles. The evaluation of dose uniformity and motion tolerance under multiple conditions, despite using a simple system, could be useful for the treatment with motion in layer-stacking irradiation.

## Supplementary Information

Below is the link to the electronic supplementary material.

Supplementary file1 (PDF 334 KB)

Supplementary file2 (PDF 359 KB)

Supplementary file3 (PDF 398 KB)

## Data Availability

The data that support the findings of this study are available upon reasonable request from the authors.
