# Supplementary material for: Evaluation of dose distributions and respiratory motion tolerance for layer-stacking conformal carbon-ion radiotherapy
Source: Radiol Phys Technol. 2024 Nov 14;18(1):3–16. doi: 10.1007/s12194-024-00847-1 (PMC11876241; doi:10.1007/s12194-024-00847-1)
Supplement: Supplementary file 1 — Supplementary file1 (PDF 334 KB) [file 12194_2024_847_MOESM1_ESM.pdf]

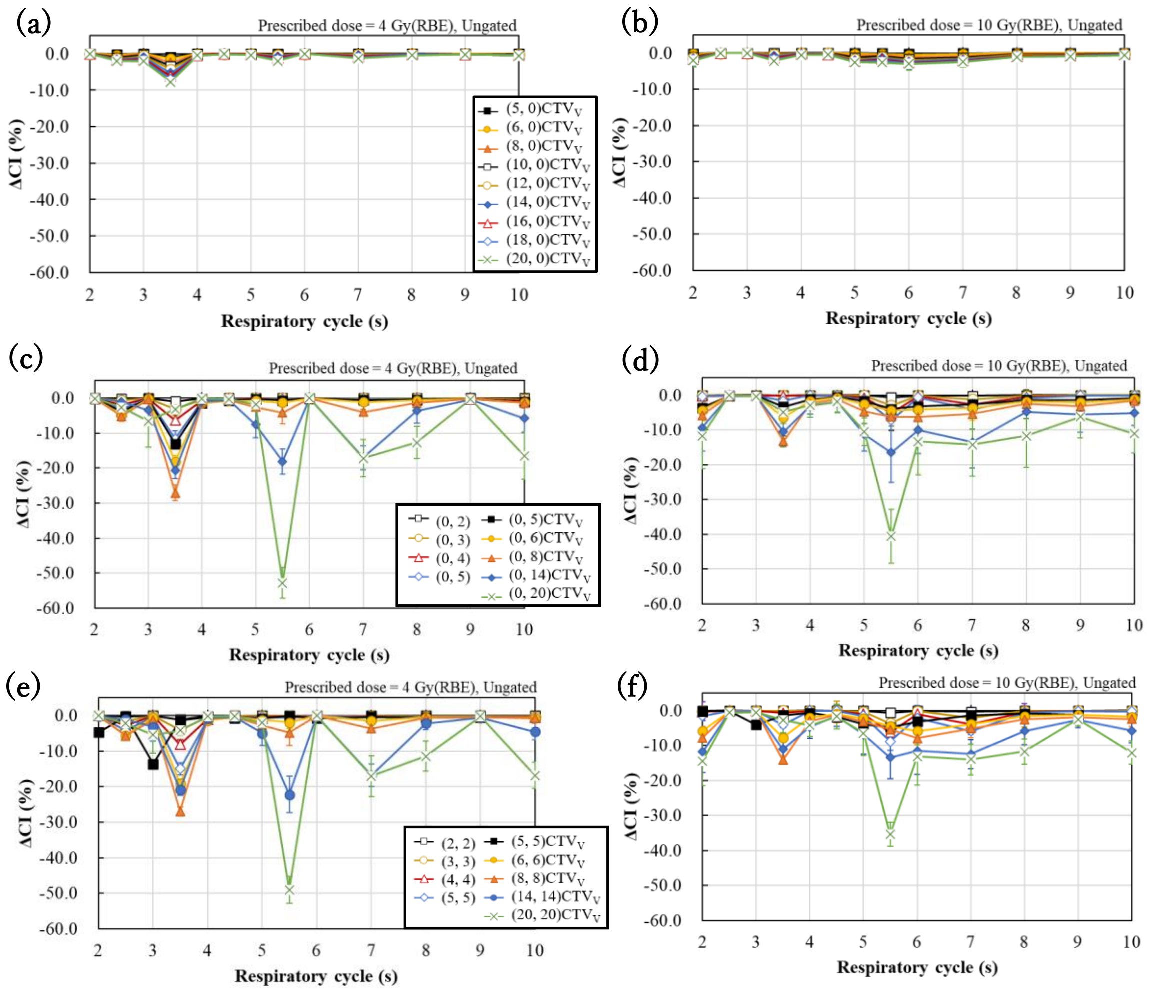

Supplementary Fig. 1 ΔCI for the respiratory cycle at prescribed doses of 4 and 10 Gy (RBE) in ungated condition. The worst ΔCI values for respiratory cycles in (a), (c), and (e) and (b), (d), and (f) are shown as ΔCI_{worst} values in Figs. 7(a) and (b) respectively. Figs. 6(e) and (f) and Supplementary Figs. 1(e) and (f) are identical
