# Supplementary material for: Evaluation of dose distributions and respiratory motion tolerance for layer-stacking conformal carbon-ion radiotherapy
Source: Radiol Phys Technol. 2024 Nov 14;18(1):3–16. doi: 10.1007/s12194-024-00847-1 (PMC11876241; doi:10.1007/s12194-024-00847-1)
Supplement: Supplementary file 2 — Supplementary file2 (PDF 359 KB) [file 12194_2024_847_MOESM2_ESM.pdf]

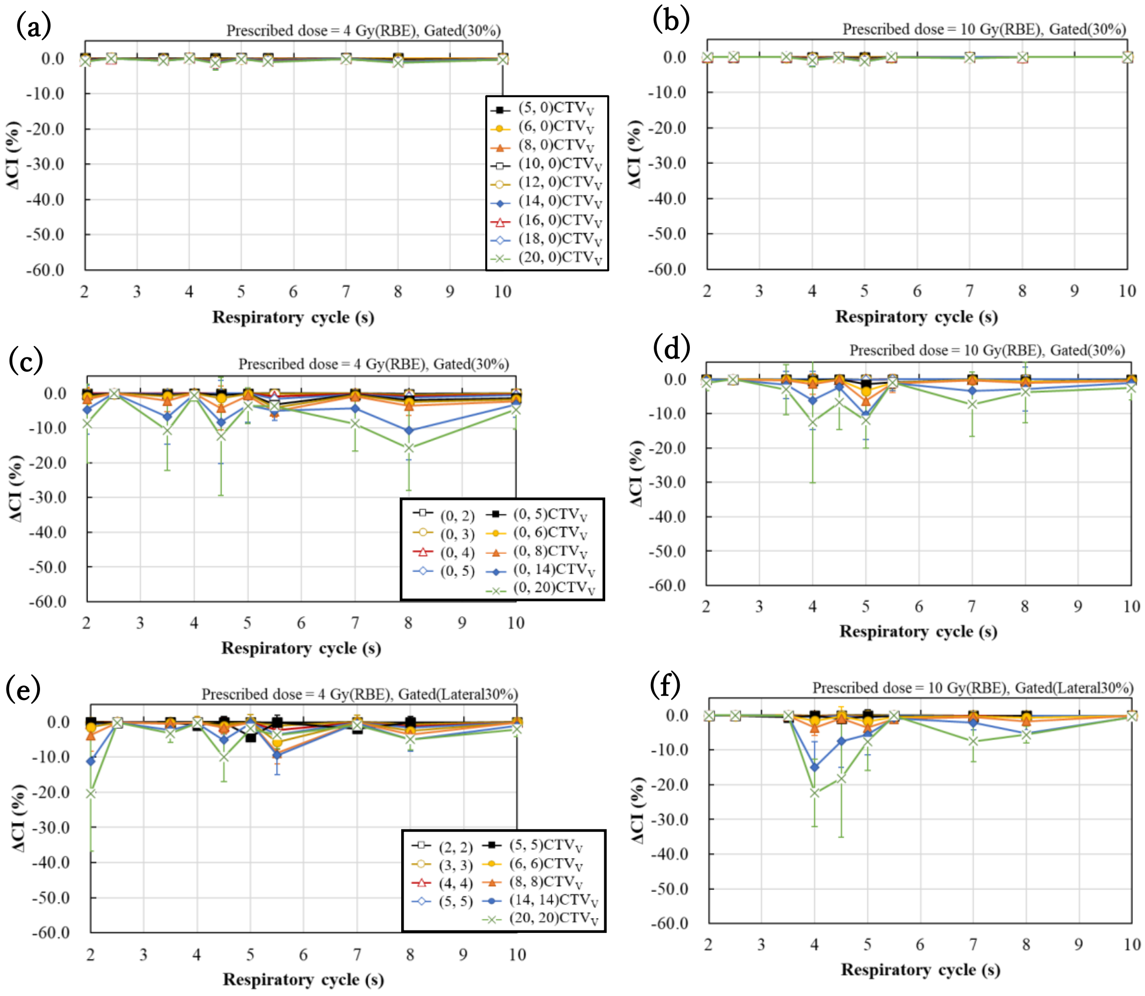

Supplementary Fig. 2 ΔCI for the respiratory cycle at prescribed doses of 4 and 10 Gy (RBE) in gated condition. The worst ΔCI values for respiratory cycles in (a), (c), and (e) and (b), (d), and (f) are shown as ΔCI_{worst} in Figs. 7(c) and (d) respectively
