# Supplementary material for: Evaluation of dose distributions and respiratory motion tolerance for layer-stacking conformal carbon-ion radiotherapy
Source: Radiol Phys Technol. 2024 Nov 14;18(1):3–16. doi: 10.1007/s12194-024-00847-1 (PMC11876241; doi:10.1007/s12194-024-00847-1)
Supplement: Supplementary file 3 — Supplementary file3 (PDF 398 KB) [file 12194_2024_847_MOESM3_ESM.pdf]

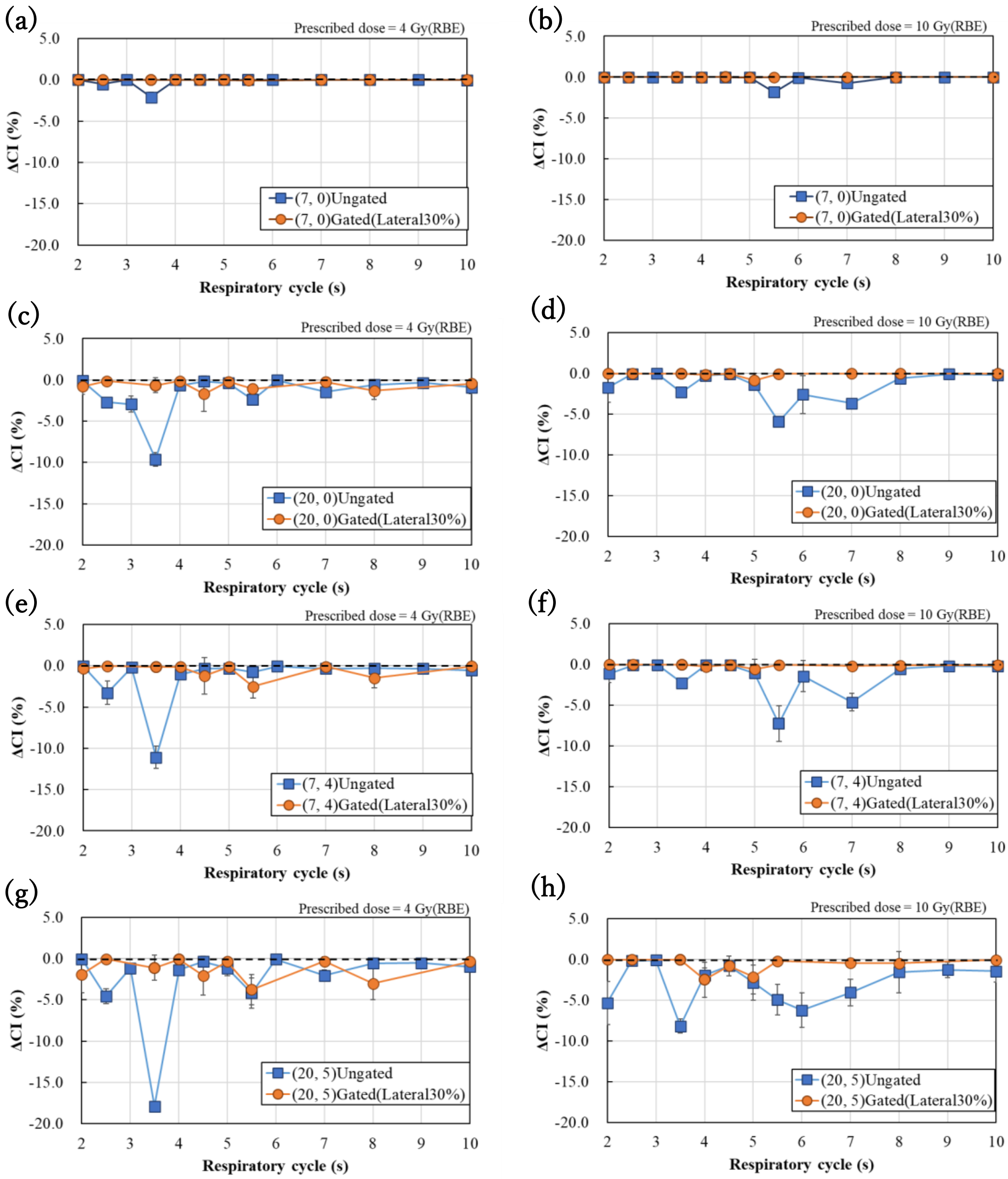

Supplementary Fig. 3 ΔCI for the respiratory cycle at prescribed doses of 4 and 10 Gy (RBE). The numbers in parentheses in the legend indicate the motion amount during irradiation in the lateral and proximal directions. ex) (Lateral, Proximal) = (7, 0). The worst ΔCI for respiratory cycles in (a), (c), (e), and (g), and (b), (d), (f), and (h) are shown as ΔCI_{worst} values in Figs. 7(e) and (f) respectively
